# The Development of Naringin for Use against Bone and Cartilage Disorders

**DOI:** 10.3390/molecules28093716

**Published:** 2023-04-25

**Authors:** Juwen Gan, Xiaolan Deng, Yonghong Le, Jun Lai, Xiaofei Liao

**Affiliations:** 1 Department of Pulmonary and Critical Care Medicine, Ganzhou People’s Hospital, Ganzhou 341000, China; 2 Department of Pharmacy, Haikou Affiliated Hospital, Xiangya School of Medicine, Central South University, Haikou 570208, China; 3 Department of Pharmacy, Ganzhou People’s Hospital, Ganzhou 341000, China

**Keywords:** naringin, osteoporosis, intervertebral disc degeneration, osteoarthritis, rheumatoid arthritis, osteogenic differentiation, bone tissue engineering

## Abstract

Bone and cartilage disorders are the leading causes of musculoskeletal disability. There is no absolute cure for all bone and cartilage disorders. The exploration of natural compounds for the potential therapeutic use against bone and cartilage disorders is proving promising. Among these natural chemicals, naringin, a flavanone glycoside, is a potential candidate due to its multifaceted pharmacological activities in bone and cartilage tissues. Emerging studies indicate that naringin may promote osteogenic differentiation, inhibit osteoclast formation, and exhibit protective effects against osteoporosis in vivo and in vitro. Many signaling pathways, such as BMP-2, Wnt/β-catenin, and VEGF/VEGFR, participate in the biological actions of naringin in mediating the pathological development of osteoporosis. In addition, the anti-inflammatory, anti-oxidative stress, and anti-apoptosis abilities of naringin also demonstrate its beneficial effects against bone and cartilage disorders, including intervertebral disc degeneration, osteoarthritis, rheumatoid arthritis, bone and cartilage tumors, and tibial dyschondroplasia. Naringin exhibits protective effects against bone and cartilage disorders. However, more efforts are still needed due to, at least in part, the uncertainty of drug targets. Further biological and pharmacological evaluations of naringin and its applications in bone tissue engineering, particularly its therapeutic effects against osteoporosis, might result in developing potential drug candidates.

## 1. Introduction

Bone and cartilage disorders, the leading causes of musculoskeletal disability, are characterized by the destruction of bone and cartilage, as manifested by imbalanced homeostasis, increased inflammatory responses, and dysregulated osteoimmunology [1]. The prevalence of bone and cartilage disorders has increased significantly due to the aging population. This situation has posed a challenge to the economy and society worldwide. In particular, bone infection, a difficult-to-treat disease, may enhance the ratio of treatment failure by up to 20–30% [2]. Although promising experimental research and the exploration of clinical diagnosis and therapeutic management techniques are currently underway, there is no absolute cure for all bone and cartilage disorders. An effective clinical treatment strategy for bone and cartilage disorders is essential.

Researchers are interested in the potential efficacy of synthetic and/or natural compounds against bone and cartilage disorders [3,4]. Tofacitinib, a Janus kinase inhibitor, is a disease-modifying anti-rheumatic drug (DMARDs) and shows therapeutic effects against rheumatoid arthritis (RA). A recent study shows that tofacitinib (5 mg and 10 mg BID) exhibits a consistent safety profile and sustained efficacy for patients with RA [5]. Tocilizumab, a non-tumor necrosis factor inhibitor (TNFi) biologic agent, has a satisfactory efficacy against RA, with a low risk of cardiovascular events [6]. The chicken embryo tissue hydrolysate (CETH) dose-dependently reverses the morphological changes of RA induced by Freund’s full adjuvant in rats [7]. Several natural chemicals and synthetic nanoparticular compounds have been reviewed for their therapeutic effects against osteoarthritis (OA) [8].

**Figure 1:**
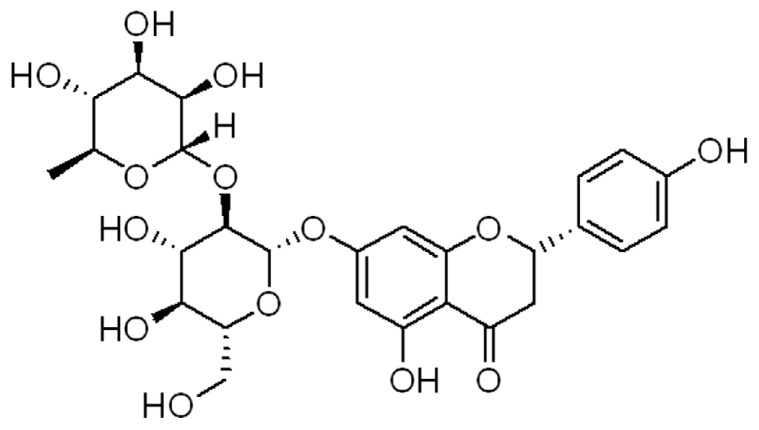
The chemical structure of naringin.

Flavonoids are polyphenolic compounds and a source of effective chemicals in plants and nutraceuticals consumed by humans. Studies have reported that these effective compounds exhibit various pharmacological activities by modulating certain enzymes. For example, genistein may exhibit estrogen-like effects to protect against many diseases, such as cancers, osteoporosis (OP), and OA [9]. Icariin shows promising characteristics in biomedicine and bone/cartilage tissue engineering [10]. Naringin (4′, 5,7-trihydroxy flavanone-7-rhamnoglucoside, Figure 1) is a flavonoid glycoside that is found in the extract of citrus fruits [11] and rhizoma drynariae [12]. Naringin possesses several pharmacological effects, including anti-inflammation, anti-oxidation, anti-cancer, anti-bacteria, liver protection, and bone/cartilage protection [13,14].

The pharmacokinetic profiles and bioavailability of naringin have been comprehensively discussed recently [15,16]. Briefly, naringin can be poorly absorbed in the gastrointestinal tract and converted to its aglycone form, naringenin, by gut microorganisms [17]. The bioavailability of naringin by oral administration is about 5–9% [18], and the Cmax value is about 5.5 h [19]. The distribution of naringin includes the trachea, gastrointestinal tract, lungs, liver, and kidneys. After administration, naringin undergoes oxidation, demethylation, glucuronidation, and sulfation [20]. Naringin is a nontoxic bioactive natural compound. It has been reported that the oral administration of naringin at a dose of 200 mg/kg in humans exhibits no obvious side effects [21].

Naringin has been used in traditional Chinese medical regimens to treat osteoporosis [22]. Naringin may mediate the intracellular signaling pathways that are important for the development of bone and cartilage. Currently, the applications of naringin in bone are particularly strongly associated with the therapeutic management of OP and the pro-osteogenic differentiation of mesenchymal stromal stem cells (MSCs). The beneficial effects of naringin are also seen in its contribution to the field of tissue engineering [23]. The potential therapeutic effects of naringin might be associated with its biological actions on intracellular targets. In this review article, we describe the protective effects of naringin against bone and cartilage disorders, including OP, OA, intervertebral disc degeneration (IDD), RA, femoral head (FH) diseases, bone and cartilage tumors, and tibial dyschondroplasia (TD).

## 2. The Protective Activities of Naringin against Osteoporosis

OP is characterized by a reduction in bone mineral density (BMD), a deterioration of the bone microstructure, and the degradation of matrix protein. These pathological changes may lead to an increase in bone fragility and fracture risk. OP causes a reduction in quality of life and places a financial burden on patients and society [24]. The causes of OP might be associated with the hypofunction of osteoblasts and the hyperfunction of osteoclasts. Therefore, improving osteoblast biofunctions and attenuating osteoclast activity can be an effective therapeutic strategy for OP treatment. The clinically approved drugs regulate the remodeling of bone in ways that increase BMD, strengthen bones, and decrease fracture risks. Anti-resorptive drugs, such as bisphosphonates, calcitonin, and denosumab, improve BMD and restore bone strength; as a result, they reduce fracture risk by inhibiting bone resorption [25]. Osteoanabolic drugs, such as teriparatide, stimulate new bone formation and improve bone strength and bone microarchitecture (the parathyroid hormone restores bone quality to levels equal to the control group, at least in a rodent model) [26]. Both blocking bone resorption and stimulating new bone formation are effective strategies for OP treatment.

The current OP drug therapies have been reviewed in detail by Reid et al. [27]. Anti-resorptives, including bisphosphonates, are the first line of treatment against osteoporosis. Denosumab, another first-line anti-osteoporotic drug, is a monoclonal antibody against RANKL and acts as an anti-resorptive, similar to bisphosphonates. The osteoanabolic drugs teriparatide and abaloparatide, two different synthetic peptide analogs of human PTH, bind and activate the parathyroid hormone-1 receptor (PTH1R), stimulating bone formation [28]. Statins, the inhibitors of hepatic hydroxy-methylglutaryl coenzyme A (HMG-CoA) reductase, have been reported to stimulate the expression of bone morphogenetic protein-2 (BMP-2), which is a regulator of osteogenic differentiation. Statins may produce anabolic effects on bone. However, the administration of statins can induce several side effects, such as weakness, headache, and muscle problems [29]. Thus, the therapeutic inhibition of HMG-CoA reductase expression can be a potential strategy for developing bone-inductive agents.

Naringin acts as an inhibitor of HMG-CoA reductase. In addition, naringin may increase bone formation, as shown by increased cell viability and enhanced ALP activity in UMR-106 cells [30]. The positive effects of naringin on bone in rodents, particularly in females in most research, have been demonstrated. Naringin or hesperidin, and a combination of both, have been linked to the accrual and maintenance of BMD and the structure and strength of bone [31,32]. A recent study indicates that the maternal consumption of naringin and hesperidin during pregnancy and lactation shows transient effects on the BMD in trabecula and the structure of bone at the proximal tibia in female CD-1 mice offspring after weaning. However, these biological effects cannot influence the skeletal integrity and strength of the tibia, femur, and LV in adulthood [33].

### 2.1. Osteogenic Differentiation Induction

Naringin can effectively enhance the proliferative activity of MC3T3-E1 cells and promote the differentiation of osteoblasts, as shown by the increased activity of ALP [34]. In human bone marrow mesenchymal stem cells (BMSCs), naringin has been demonstrated to promote proliferation and osteogenic differentiation, as indicated by an increased expression of ALP, OPN, OCN, and COL1A2 [35]. Another study shows that naringin may promote osteoblast differentiation and suppress adipocyte formation by mediating miR-20a/PPARγ signaling in BMSCs. Specifically, the naringin-increased expression of miR-20a targets the degradation of PPARγ, which may induce BMSCs to differentiate into adipocytes [36]. Fish-derived collagen films fabricated with genistein, icariin, and naringin may improve the proliferation and differentiation of human MSCs by upregulating the expression of RUNX-2, Collagen I, c-Fos, and TGFβ1/Smad3. In addition, the mechanical properties, solubility, and in vitro biodegradation of the fabricated collagen films were also improved [37].

Human periodontal ligament stem cells (hPDLSCs) may differentiate into osteoblasts, osteoclasts, fibroblasts, and cementum cells. Naringin, at a dose of 1 μM, can promote the proliferation and differentiation of hPDLSCs into osteoblasts, as indicated by upregulating bone-related factors, such as RUNX2, COL1A2, OPN, and OCN [38]. However, the limited lifespan of hPDLSCs restricts their application [39]. The knock-in of Bmil may enhance the cell cycle, cell replication, and stemness of hPDLSCs. Interestingly, naringin induces the osteogenic differentiation of hPDLSC-Bmil cells by stimulating the expression of the ERK1/2 signaling pathway [40].

Similarly, naringin ameliorates the H_2_O_2_-induced inhibition of osteogenic differentiation by activating the Wnt/β-catenin signaling pathways in human adipose-derived stromal cells (ADSCs) [41]. Consistently, naringin rescues H_2_O_2_-inhibited β-catenin and cyclin D1 expression, stimulates ALP activity, increases RUNX2 and OSX expression, and promotes osteogenic differentiation by inhibiting oxidative stress in human adipose-derived mesenchymal stem cells (hADMSCs) [42]. Similarly, human amniotic fluid-derived stem cells (hAFSCs) can also produce osteogenic cells, increase the expression of RUNX2, OPG, OPN, and Collagen I, and decrease the expression of RANKL by upregulating the activity of the BMP4 and Wnt/β-catenin signaling pathways [43].

The directional migration of the MSCs and osteogenesis contribute to bone fracture healing. In addition, MSCs may produce a series of cytokines, growth factors, and chemokines by autocrine and paracrine, affecting bone fracture healing. Naringin can stimulate MSC migration and induce differential chemokine secretion of the C-X-C motif chemokine-5 (CXCL-5), CXCL-6, and the C-C motif chemokine-20 (CCL-20) by activating the Ras signaling pathway [44]. Another study demonstrated that naringin might promote the osteogenic differentiation of human BMSCs by increasing the phosphorylation of ERK, as indicated by the upregulated expression of RUNX-2, OCN, COL1, and osterix (Osx). The inductive effect of naringin may be abolished by U0126, which is a specific inhibitor of ERK [45].

### 2.2. Osteoclast Formation Inhibition

Increased osteoclast formation and bone resorption contribute to the development of osteolytic bone diseases, such as OP. The receptor activator of the nuclear factor-κB (NF-κB) ligand (RANKL) contributes to the formation and activation of osteoclasts. The association of RANKL with RANK triggers the intracellular signaling pathways, such as NF-κB, AKT, MAPKs, and Ca2+/calmodulin (CaM)-dependent kinase [46]. Naringin can suppress RANKL-induced NF-κB signaling by inhibiting RANKL-regulated IκBα degradation, perturbing osteoclast formation and bone resorption. Specifically, naringin downregulates the expression of the osteoclast gene biomarkers, such as cathepsin k, calcitonin receptor, and TRACP, in a dose-dependent manner [47].

In lipopolysaccharide (LPS)-induced rat alveolar bone resorption models, naringin may increase the BMD value, decrease the osteoclast number, and increase the production of the non-calcified bone-like matrix, demonstrating osteogenic effects [48]. Retinoic acid (ReA) is used to treat psoriasis and nodular acne. The adverse reactions of ReA include OP. In ReA-induced rat OP, naringin can reduce the serum levels of alkaline phosphatase (ALP) and improve the bone weight coefficient, the length, and diameter of the bone, the content of bone ash, calcium, and phosphorus, and promote new bone formation [49]. Naringin consistently increases the number of trabeculae, improves trabecular bone structure, and increases the bone mineral density of the femur neck in ReA-induced rat OP. The possible mechanism of naringin in protecting against OP might be associated with the upregulation of ALP and PTH1R expression and the downregulation of bone resorption-related proteins, such as TRAP, RANKL, and RANK [50] (Table 1).

Osteoclast-associated bone resorption plays a critical role in aseptic loosening after joint arthroplasty. The regulation of osteoclast maturation is associated with the balance of RANKL and OPG. Naringin effectively suppresses osteoclastogenesis and wear-particle-stimulated osteolysis by increasing Wnt/β-catenin signaling-mediated OPG expression. However, naringin does not affect RANKL expression in fibroblasts [67]. Consistently, naringin, administered by intraperitoneal injection for 7 days at doses of 50 μg/kg and 100 μg/kg, respectively, may significantly suppress polymethyl methacrylate (PMMA)-induced osteolysis and aseptic loosening, which is promoted by wear-particle-induced inflammatory cytokines. In an in vitro study, naringin effectively inhibited osteoclastogenesis and osteoclast maturity functions [68]. Another study indicated that, at a dose of 10–100 μg/mL, naringin might decrease PMMA-induced TRAP activity, inflammatory responses, osteoclastogenesis, and bone resorption. Administered at a dose of 300 mg/kg by oral gavage for 30 days, naringin may ameliorate periprosthetic bone resorption [69].

### 2.3. The Underlying Mechanisms of Naringin in Protecting against Osteoporosis

**Figure 2:**
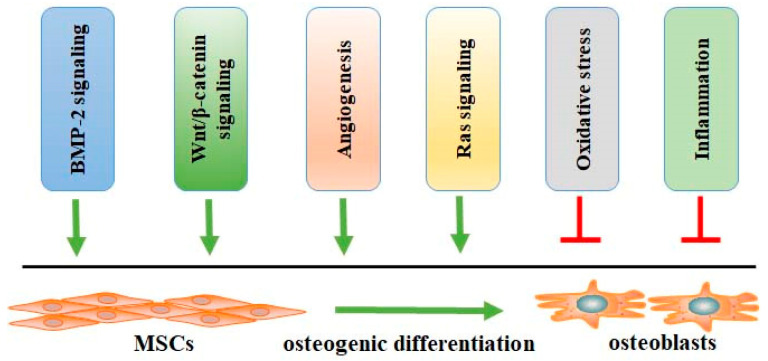
The factors affecting the processes of osteogenic differentiation of MSCs to osteoblasts. Many factors, such as BMP-2 signaling, Wnt/β-catenin signaling, angiogenesis, Ras signaling, oxidative stress, and inflammation, may affect osteogenic differentiation. Abbreviations: BMP-2, bone morphogenetic protein-2; MSCs, mesenchymal stromal stem cells.

**Figure 3:**
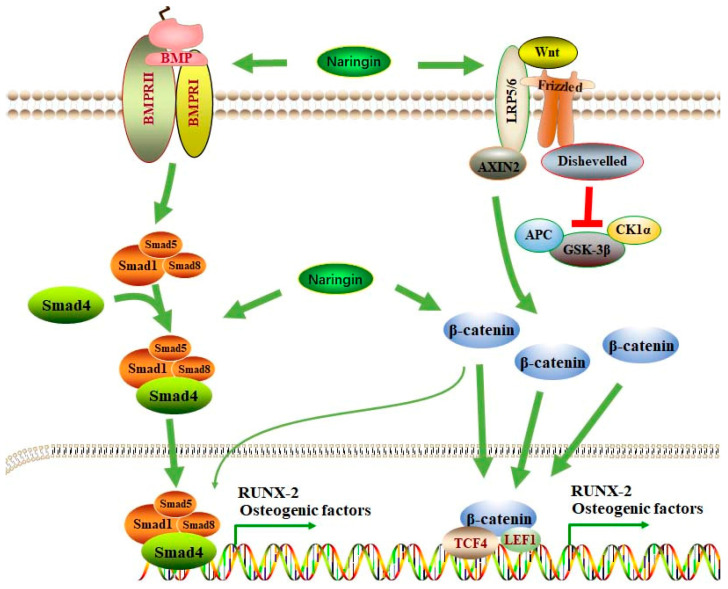
BMP-2 and Wnt/β-catenin signaling pathways participate in the pharmacological activity of naringin in mediating osteogenic differentiation. BMP-2 may interact with its receptors, such as BMPRII and BMPRI; this is followed by the activation of Smad1/5/8, which recruits Smad4, forming a complex and entering the nucleus for the transcriptional mediation of osteogenic factors, such as RUNX-2. After being activated by Wnt, β-catenin is stabilized and translocated to the nucleus for the transcriptional mediation of osteogenic factors. Activated β-catenin may also stimulate the BMP-2/Smad signaling pathway to induce osteogenic differentiation. Naringin can stimulate the BMP-2/Smad and Wnt/β-catenin signaling pathways to promote osteogenic differentiation. Abbreviations: BMP, bone morphogenetic protein; BMPRs, BMP receptors; SMAD: suppressor of mothers against decapentaplegic; Runx-2, Runt-related transcription factor-2; LRP, LDL receptor-related protein; Axin2, axis inhibition protein 2; APC, adenomatous polyposis coli; CK1α, casein kinase 1α; TCF-4, transcription factor 4; LEF1, lymphoid enhancer-binding factor 1.

Emerging evidence has reported that many factors, such as BMP-2, Wnt/β-catenin, angiogenesis, and oxidative stress, may affect the biological processes of osteogenic differentiation (Figure 2), which is the potential target for the therapeutic management of OP. Naringin promotes osteogenic differentiation, as shown by the increased expression of ALP, bone sialoprotein (BSP), and RUNX-2, as well as increased calcium deposits and a decreased expression of PPARγ2 in rat BMSCs [70]. BMP-2 mediates cell proliferation and differentiation by binding with BMP receptors (BMPRs) (Figure 3), such as BMPR-1A and BMPR-1B, and activating the BMPR signaling pathway. The activation of BMP-2/BMPR-1A may initiate the differentiation of BMSC into adipocytes. In contrast, BMP-2/BMPR-1B signaling activation may result in osteogenesis and bone formation [71]. Naringin and naringin-containing *Drynaria fortunei* (Gusuibu) may block the interaction between BMP-2 and BMPR-1A and enhance the binding of BMP-2 to BMPR-1B, promoting osteoblast differentiation [72]. However, some studies indicate that BMPR-1A is essential for osteogenesis and chondrogenesis, and BMPR-1B inhibits hypertrophic differentiation. BMPR-1B activation facilitates chondrogenesis over hypertrophy [73]. These findings suggest that differentiating the competitive interaction of BMP-2 with their receptors may govern the differentiation of BMSCs.

The BMP-2 signaling pathway in the demineralized bone matrix (DBM) is also involved in osteogenic differentiation during the repairing of bone fractures. Naringin may act as an activator of the BMP-2 promoter and increase bone formation. In rabbits with bone/collagen matrix grafts, naringin in the collagen matrix increases new bone by 284% and 490% more than the autogenous endochondral bone grafts alone and a collagen matrix alone, respectively [30,74]. The inductive activity of BMP-2 in osteogenic differentiation is also regulated by the PI3K/AKT, c-Fos/c-Jun, and AP-1 signaling pathways. Mutations of p85, Akt, c-Fos, and c-Jun may block the potentiating actions of naringin on the expression of BMP-2 in osteoblasts [75]. The notch signaling pathway regulates cell functions, such as proliferation, differentiation, and cell-fate decisions. Notch reception activation may produce γ-secretase-cleaved NICD, which is translocated into the nucleus for the transcriptional regulation of target genes, such as HES [76]. The importance of notch signaling in bone formation has been demonstrated [77]. It is reported that notch signaling is activated and regulates BMP-2/DLX3-mediated osteogenic differentiation in dental follicle cells [78].

Wnt/β-catenin signaling has been associated with osteogenesis [79] (Figure 3). Naringin may promote the phosphorylation of the Ser552 residue on β-catenin, leading to the stabilization and nuclear translocation of β-catenin and the upregulation of ALP, RUNX-2, and COL1 expression in rat osteoblast-like UMR-106 cells. However, treatment with AKTi (an AKT inhibitor) or Dorsomorphin (an AMPK inhibitor) may significantly reduce the effects of naringin on β-catenin. In OVX-induced mouse OP, the osteoprotective effects of naringin are also abolished by AKTi or Dorsomorphin [80]. Sclerostin, an antagonist of Wnt/β-catenin signaling, plays an important role in the development of OP. Periostin, an upstream regulator of sclerostin, negatively regulates sclerostin expression in response to mechanotransduction by upregulating Sost expression [81]. Semaphorin 3A (Sema3A), an axonal guidance chemorepellent in the nerve system, plays a critical role in the maintenance of bone mass. Sema3A can be produced by osteoblasts and osteocytes, promoting bone formation by stimulating osteoblast differentiation through the upregulation of the Wnt/β-catenin signaling pathway and downregulation of osteoclast differentiation [82]. In unilateral sciatic neurectomy (USN)-induced rat disuse OP, naringin may increase bone formation and suppress bone resorption by activating the Wnt/β-catenin signaling pathway and increasing Sema3A expression [83].

Forkhead box C2 (Foxc2), a member of the family of forkhead transcriptional factors, has been shown to promote the osteogenic differentiation of MSCs by activating Wnt/β-catenin signaling. Indian hedgehog (IHH) is a member of the hedgehog family, and it regulates the actions of tissue patterning, skeletogenesis, and cell proliferation. A deficiency of IHH may inhibit osteoblast development [84]. Naringin can upregulate the expression of Foxc2 and promote osteogenic differentiation in BMSCs. However, the inhibition of the IHH signaling pathway may block the potentiated effects of naringin on Foxc2 expression and osteogenic differentiation [85]. It has been reported that sciatic neurectomy can decrease periostin expression, increase sclerostin, and inactivate Wnt/β-catenin signaling, leading to the deterioration of the trabecular microstructure and bone loss. Naringin may exhibit osteoprotective effects against sciatic-neurectomy-induced bone loss by suppressing sclerostin expression and activating Wnt/β-catenin signaling [86].

Estrogen and selective estrogen receptor modulators (SERMS) act primarily as anti-resorptive candidates. Phytoestrogens, such as flavonoids, have been investigated as new strategies for managing estrogen-deficient diseases, such as OP. Naringin has estrogenic agonist activity at low concentrations, as it selectively interacts with estrogen receptor β (ERβ), preventing OP development. However, at high concentrations, naringin exhibits anti-estrogenic effects [87]. Another study reports that naringin, at doses of 0.2 and 0.4 mg/g/day, can enhance the value of BMD at the distal femur, proximal tibia, and lumber spine in ovariectomized (OVX) mice. The protective activity of naringin may be abolished by ER antagonist ICI-182780 in rat osteoblast-like UMR-106 cells [88]. One study demonstrated that naringin could enhance cell viability and proliferation by increasing the translocation of ERα to the nucleus for transcriptional regulation in MC3T3-E1 cells. In addition, the Erα-specific inhibitor, methylpiperidinopyrazole, can abrogate the naringin-stimulated expression of ERα and ALP, suppressing bone healing and mass in ICR mice [89]. A meta-analysis of the protective effects of naringin against postmenopausal OP in OVX rats found that naringin may promote bone formation [90]. Naringin exhibits protective activity against OVX-induced rat OP by promoting mitochondria-mediated apoptosis in osteoclasts. Specifically, naringin can decrease the expression of Bcl-2 and increase the expression of Bax, caspase-3, and cytochrome C in osteoclasts, reducing bone resorption [51] (Table 1). When combined with treadmill exercise, naringin may protect against OVX-induced rat OP, as indicated by an increased BMD, improved morphological bone indices, the upregulation of osteocalcin (OCN) expression, and the downregulation of the C-terminal telopeptides of type I collagen (CTX-1) [52] (Table 1).

Blood vessels and angiogenesis may play an essential role in orchestrating the balance between bone regeneration and resorption (Figure 3). Postmenopausal OP is associated with reduced sinusoidal and arterial capillaries in the bone marrow and decreased bone perfusion. Vascular endothelial growth factor (VEGF) can improve fracture repair by stimulating angiogenesis, ossification, and bone metabolism [91]. Thus, increased angiogenesis can be an effective strategy for the therapeutic management of OP. Recently, the effects of naringin on vascular endothelial cells (VECs) in OVX-induced rat OP have been explored. Naringin may suppress the expression of GRP78, CHOP, caspase-12, caspase-3, and caspase-9, increase the production of NO, inhibit cell apoptosis, and improve the values of BMD, leading to the amelioration of OP by promoting angiogenesis [92]. Similarly, it is shown that naringin may induce angiogenesis in OVX-induced rat OP by activating the expression of VEGF and VEGFR-2. Specifically, naringin can increase the vessel number and larger vessel area and improve bone repair [93].

Oxidative stress is associated with a decline in tissue and organ functions (Figure 3). The redox homeostasis in cells is orchestrated by ROS generation and scavenging. Excessive ROS production may induce biological damage to the DNA, proteins, and lipids. The antioxidants include superoxide dismutase (SOD), glutathione (GSH), catalase (CAT), and glutathione peroxidase (GPX) [94]. Oxidative stress is involved in diabetes-associated bone diseases. Moreover, 60Co γ-radiation induces acentric fragments, chromatid and chromosome breaks, dicentrics, and exchanges. These aberrations in mouse bone marrow cells can be significantly suppressed by naringin (2 mg/kg) by scavenging free radicals [95]. Naringin may also reduce chromosome aberrations and reduce the frequency of micronucleated polychromatic (MPCE) and normochromatic (MNCE) in mouse bone marrow [96]. Both glucocorticoid (GC) and inflammatory bowel disease (IBD) can induce bone loss. Clinically, OP and bone fractures can occur at high rates in patients with IBD. Naringin exhibits osteoprotective effects against bone loss in GC-treated IBD rats by reducing oxidative stress and promoting bone formation [53] (Table 1). In addition, naringin promotes the proliferation and differentiation of osteoblasts by upregulating the PI3K/AKT/mTOR signaling pathway and activating autophagy. In GC-induced rat OP, naringin can increase BMD, improve bone morphology parameters, and upregulate the expression of autophagy-related factors [34]. In streptozotocin (STZ)-induced rat diabetes, naringin exhibits anti-oxidative effects in the bone marrow of the femur, increases the BMD and bone mineral content (BMC) of the distal femur and proximal tibia, decreases the number of adipocytes and TRAP-positive cells, and upregulates the expression of OCN. These findings indicate that naringin exhibits protective effects against diabetes-associated OP by increasing osteoblastogenesis and decreasing osteoclastogenesis and adipogenesis by inhibiting oxidative stress [97]. In addition, diabetes may worsen the bone indices, leading to decreased values for new bone formation, calvaria thickness, bone volume, the midline suture area, and the OCN concentration in streptozotocin (STZ)-treated mice. Naringin exhibits osteoanabolic activity and improves bone indices, promoting new bone formation [98].

The Janus-activated kinase 2 (JAK2)/signal transducer and the activator of transcription 3 (STAT3) signaling play a critical role in the pathogenesis and progression of OP. The activation of the JAK2/STAT3 signaling may stimulate the expression of RANKL and promote the differentiation of osteoclasts [99]. Another study also supports the idea that RANKL promotes osteoclastogenesis by inactivating the Akt and JAK2/STAT3 signaling pathways [100]. Naringin may significantly improve BMSC viability and osteogenic differentiation by suppressing JAK2/STAT3 signaling. In OVX-induced rat postmenopausal OP, naringin exhibits similar effects to AG490 (an inhibitor of JAK2/STAT3 signaling) to improve the bone parameters from dual-energy X-ray absorptiometry and micro-CT and suppresses the development of OP [101]. These results consistently support the finding that naringin ameliorates OVX-induced rat bone loss, increases the expression of osteocalcin, and promotes the osteogenic differentiation of BMSCs [102].

### 2.4. Potential Applications of Naringin in Bone Tissue Engineering

Titanium (Ti) and its alloys have demonstrated good biocompatibility and mechanical characteristics in clinical settings. However, Ti-implant-associated infection and tumor recurrence have challenged their application in osteosarcoma resection [103]. Zinc oxide nanoparticles (ZnO NPs), 3-carboxyphenylboronic acid (PBA), and naringin (NG) are modified to be ZnO-PBA-NG NPs, which are then immobilized on Ti substrates. This functional Ti substrate has pH-responsive characteristics and triggers the release of naringin due to the presence of bacterial infections and an acidic environment. In addition, this Ti substrate may induce oxidative stress to damage the bacterial biofilm and membrane, promote osteosarcoma cell apoptosis by stimulating the ERK signaling pathway, and stimulate osteoblast proliferation and differentiation [104].

At overly high concentrations, naringin may exhibit harmful effects on cells, while very low concentrations may be ineffective. The prevention of burst release and the facilitation of the controlled release of naringin may benefit cells. A hybrid depot of a naringin-loaded microsphere/sucrose acetate isobutyrate was prepared to control the release of naringin and prevent burst release, promoting the new bone formation rate [105]. A multifunctional mineralized collagen (Col) coating on Ti through metal–organic framework (MOF) nanocrystals was designed to control the release of naringin (NG). This substrate of Col/MOF/NG may induce osteogenic differentiation by stimulating the expression of BMP-2 and Sema3A in MSCs, as indicated by the upregulation of Collagen I and RUNX2 [106]. Another study reported that micro-Ti is covered with naringin (NA), chitosan, and gelatin multilayers, forming LBL(NA)-coated Ti. In an in vitro study, LBL(NA)-coated Ti exhibited a sustained release of naringin. In addition, LBL(NA)-coated Ti may promote the expression of ALP, RUNX-2, OCN, OPN, OPG, and COL I and suppress the expression of CTSK, NFAT, TRAP, and VATP, indicating the induction of osteoblastogenesis and the inhibition of osteoclastogenesis [107].

Additionally, researchers have explored the possibility of incorporating naringin into the electrospun nano-scaffold containing poly(ε-caprolactone) (PCL) and poly(ethylene glycol)-block-poly(ε-caprolactone) (PEG-b-PCL). Significantly, the burst release of naringin is reduced [108]. The electrospinning of the PLGA, PLLA, and PDLLA solutions with naringin, producing a naringin-loaded fiber mesh, may increase osteogenic differentiation and cellular proliferation and decrease the burst release of naringin [109]. A nanocomposite hydrogel, comprising the liposomal building blocks of naringin-loaded salmon-derived lecithin and the embedding of gelatin methacryloyl macro-sized hydrogels, was synthesized, and it may control the release of naringin and improve the characteristics of the hydrogel matrix [110].

One study reported the preparation of naringin (NG)/gelatin microspheres (GMs)/nanohydroxyapatite (nHA)/silk fibroin (SF) scaffolds. NG/GMs/nHA/SF scaffolds demonstrated good biocompatibility and biomechanical strength, enhanced the adhesion and proliferation of BMSCs, and increased the formation of calcium nodules, promoting osteogenic differentiation [111]. An NG/SF/HA scaffold was fabricated to repair bone defects, and it exhibits osteogenic and angiogenic properties. Specifically, naringin may facilitate the growth of human umbilical-cord-derived mesenchymal stem cells (hUCMSCs) in the SF/HA scaffold by enhancing the PI3K/AKT signaling pathway [112]. Naringin poly lactic-co-glycolic acid (PLGA) microspheres adhering to the SF/HA scaffold may sustain the release of naringin. The NG/PLGA/SF/HA scaffold has been demonstrated to promote the osteogenic differentiation of BMSCs by activating the Notch signaling pathway, as shown by the increased expressions of ALP, RUNX-2, BMP2, and OCN and increased calcium deposition [113]. Another study indicates that nHA/collagen (COL)/NG also exhibits desirable biocompatibility and promotes the osteogenic differentiation of BMSCs, as shown by the increased expressions of ALP, BMP2, OCN, and OPN and the increased number of calcium nodules. In an in vivo study, the nHA/COL/NG scaffold effectively repaired skull defects and exhibited great potential in bone tissue engineering [114].

Inflammatory responses affect the outcome of bone biomaterial implantation. Specifically, inflammation may induce the formation of a fibrous envelope, inhibiting the interaction of bone cells with the implant materials and promoting implantation failure. Ideally, the bone materials will induce osteogenic proliferation and differentiation and attenuate the inflammatory responses [115]. It has been reported that loading naringin into β-cyclodextrin-modified mesoporous bioactive glass nanoparticles (NG@CD-MBG) may promote macrophages to induce M2 polarization, inhibit inflammatory responses, induce osteogenesis, and suppress osteoclastogenesis. However, the over-transition of M1 to M2 may inhibit the activity of osteoclasts, leading to the delayed resorption of implant materials and old bone. Thus, pathological fibrosis and delayed bone healing may arise. NG@CD-MBG may effectively control the release of naringin, benefiting tissue regeneration [116]. A further study shows that naringin can rescue the TNFα-stimulated expression of p-IκBα and nuclear p65 and enhance the levels of RUNX-2 and Osx in BMSCs, inhibiting the NF-κB signaling pathway and promoting osteogenic differentiation [117].

## 3. The Protective Activities of Naringin against Intervertebral Disc Degeneration (IDD)

Lower back pain, a common and complicated disease, is closely associated with disability. Intervertebral disc degeneration (IDD) contributes to the development of lower back pain. More than 10% of 50-year-old and 60% of 70-year-old people have severe IDD [118]. Various factors, such as aging, immune dysregulation, inflammation, metabolic disorders, excessive mechanical loads, and insufficient nutritional supply, may promote the pathological development of IDD [119]. However, the potential molecular mechanism of IDD-associated lower back pain remains unclear. The intervertebral disc (IVD), an avascular connective tissue, is constituted by a central nucleus pulposus (NP), a peripheral annulus fibrosus (AF), and a cartilage endplate (CEP). ECM acts as the microenvironment for IVD cells, and it mediates the metabolism and functions of IVD cells. The pathological changes in IDD may involve a decreased supply of nutrition, the alterations of ECM components, and the apoptosis of IVD cells [120].

Natural compounds may be effective against IDD and low back pain. At a dose of 20 μg/mL, naringin may increase the proliferative activity of NP cells. In addition, naringin may suppress the production of TNFα, enhance the expression of BMP-2, collagen II, aggrecan, and SOX6, and inhibit the expression of MMP-3 in NP cells [54] (Table 1). It has been shown that inflammation plays a critical role in the development of IDD [121]. Pro-inflammatory cytokines, such as IL-1β and IL-6, may promote the expression of MMPs and ADAMTSs, which can induce the degradation of collagen II and aggrecan and contribute to the pathological development of IDD [122]. In IL-1β-treated human NP cells, naringin may decrease the expression of MMP-3, MMP-13, ADAMTS4, ADMATS5, IL-6, and TNFα and increase the production of collagen II and aggrecan by suppressing the NF-κB and p53 signaling pathways [55] (Table 1).

TNFα can also trigger an inflammatory cascade, which promotes mitochondrial dysfunctions and ROS generation in NP cells. Autophagy, an evolutionarily conserved stress-responsive process, can dispose of the damaged cytoplasmic organelles and transport them for their degradation in lysosomes. Autophagy contributes to the recycling of these damaged organelles for cell metabolism. The over-activation of autophagy may promote cell survival. The AMP-activated protein kinase (AMPK)/Sirt1 signaling pathway facilitates mitochondrial homeostasis by producing an autophagic effect [123]. Naringin may increase Beclin-1, the LC3-II/LC3-I ratio, and collagen II expression, decrease p62 and MMP-3 expression, and inhibit cell apoptosis by activating AMPK/Sirt1 signaling in TNFα-treated NP cells [56] (Table 1).

Additionally, naringin can enhance autophagy and suppress oxidative-stress-associated apoptosis by upregulating the AMPK signaling in NP cells. In an in vivo study, naringin was shown to ameliorate the pathological development of IDD in puncture-treated rats [124]. In cyclic stretch-treated rat AF cells, naringin consistently decreased cell apoptosis by suppressing oxidative stress and the NF-κB signaling pathway, delaying the pathological development of IDD [125]. Increased apoptosis of NP cells is one of the main pathological changes of IDD. In H_2_O_2_-treated NP-derived MSCs, naringin has exhibited protective activities against H_2_O_2_-induced oxidative stress, mitochondrial dysfunction, MMP expression, and cell apoptosis by activating the PI3K/AKT signaling pathway [57] (Table 1).

## 4. The Protective Activities of Naringin against Osteoarthritis (OA)

OA is associated with chronic inflammation, osteophyte formation, cartilage degeneration, and subchondral bone sclerosis. The global prevalence of OA has been estimated to be 23% among middle- to old-aged individuals [126]. Currently, no effective drugs are available to cure OA. Clinically, symptom relief becomes the effective strategy to treat OA. TNFα and IL-1βplay a central role in the pathological development of OA [127]. The inhibition of inflammatory responses is an effective therapeutic strategy for managing OA. Naringin can suppress the TNFα-induced inflammatory responses and ECM degradation in mouse chondrocytes by downregulating the NF-κB signaling pathway, as indicated by the decreased expression of IL-1β, iNOS, COX-2, MMP-13, and ADAMTS5. In ACLT-induced mouse OA models, naringin may significantly improve the OARSI scores and pathohistological changes and decrease the expression of catabolic factors [58] (Table 1).

The anti-inflammatory activity of naringin in LPS-treated RAW 264.7 cells and monosodium iodoacetate (MIA)-induced rat OA shows that naringin can reduce the generation of PGE2, NO, IL-6, and TNFα, protecting against OA development [59] (Table 1). Articular cartilage is an avascular tissue with limited self-repair abilities. MSCs demonstrate an ability to differentiate into cartilage cells [128]. A combination of naringin with rabbit BMSCs is effective in the repair of cartilage defects in rabbit knee joint cartilage. Mechanically, naringin/BMSCs significantly enhance the expression of TGFβ3 and SOX9, promoting chondrocyte differentiation and displaying satisfactory therapeutic effects [129]. The TGFβ/Smad signaling pathway is closely associated with the process of repairing cartilage injury. Combined with the acellular dermal matrix (ADM), naringin may significantly improve the pathological changes in the defect structures of cartilage in New Zealand rabbits by upregulating the expression of TGFβ2, TGFβ3, and SOX9 [60] (Table 1).

## 5. The Protective Activities of Naringin against Rheumatoid Arthritis (RA)

Rheumatoid arthritis (RA) is an autoimmune disease characterized by the progressive destruction of bone and cartilage due to chronic inflammation and T-cell and macrophage infiltration. No specific drugs are available to treat RA. The global prevalence of RA is estimated to be 0.5–1.0% [130]. Persistent inflammatory responses may result in synovial hyperplasia, joint deformity, and disability. Clinically, nonsteroidal anti-inflammatory drugs (NSAIDs), Janus kinase inhibitors, and disease-modifying anti-rheumatic drugs are used to improve the symptoms [131]. The pathogenesis of RA is complicated. Infection, genetics, and environmental factors contribute to RA development [132]. However, the potential molecular mechanisms that mediate the pathological development of RA remain unclear.

Naringin has demonstrated anti-inflammatory and pro-apoptotic activities in RA fibroblast-like synoviocytes (FLSs), as indicated by the decreased expression of IL-1, IL-6, and IL-8, the increased expression of caspase-3 and an increased Bax/Bcl-2 ratio, and attenuated expression of MMP-1, MMP-2, and MMP-13; it achieves this by downregulating the MAPK/ERK and PI3K/AKT signaling pathways [61] (Table 1). A combinational therapy against inflammation and arthritis has become a promising strategy. Naringin-containing combination liposomal formulations (CLFs) can significantly improve both paw edema and the arthritic score in FCA-induced rat RA models [133].

## 6. The Protective Activities of Naringin against Femoral Head (FH) Diseases

Femoral head (FH) disease, a refractory disease in bone, can be separated into invasive and non-invasive clinical groups. Skeletal trauma, such as femoral neck fracture and hip dislocation, can be included in the invasive group. Steroid- and alcohol-induced FH diseases are non-invasive [134]. The incidence of osteonecrosis of the FH (ONFH) is 2.91 cases per 100,000 person-years in Japan [135]. In Korea, the estimated yearly prevalence was 37.96/100,000 in 2006 [136]. In China, there were approximately 8.12 million cases among Chinese people aged ≥15 in 2010 [137]. Clinically, FH diseases are considered complications of glucocorticoid therapy. However, the underlying molecular mechanisms of glucocorticoid-induced FH diseases are still unelucidated. Fat embolism, vascular thrombosis, osteocyte apoptosis, and oxidative stress contribute to steroid-induced osteonecrosis in the femoral head [138]. The protective effects of naringin on steroid-induced avascular necrosis of the femoral head (SANFH) have been investigated. Naringin can protect against methylprednisolone (MPS)-induced increases in the total cholesterol and LDL/HDL ratio, decreases in the serum levels of osteocalcin, the downregulation of caspase-3 expression, and the inhibition of osteoblast differentiation by enhancing the protein expression of PPARγ, Notch, β-catenin, and p-AKT [62] (Table 1). Naringin stimulates osteogenesis and osteocyte proliferation and suppresses osteoclastogenesis and osteocyte apoptosis by activating the AKT/Bad signaling pathway. Specifically, naringin promotes Bcl-2 expression and decreases Bax and cleaved caspase-3 expression, inhibiting apoptosis in Dex-treated MLO-Y4 cells. In addition, naringin upregulates the expression of RUNX2, OPG, and collagen I and downregulates the expression of RANKL, promoting osteogenesis and inhibiting osteoclast formation [63] (Table 1).

## 7. The Protective Activities of Naringin against Bone and Cartilage Tumors

The multiple pharmacological properties of naringin suggest its beneficial effects on the therapeutic management of cancers. The preventive activities of naringin against tumor development in humans have been reported in detail [139]. The potential molecular mechanisms of naringin in counteracting tumor development may include, at least in part, the MAPK/NF-κB, TGFβ/Smad, Wnt/β-catenin, and JAK2/STAT3 signaling pathways [140]. Osteosarcoma is one of the most common types of bone cancer. The annual incidence of osteosarcoma is 3.1 per million [141]. Treatment for osteosarcoma involves surgical resection, chemotherapy, and interventional therapy. Recently, adjuvant chemotherapy has increased the survival rate, and the development of multidrug resistance has posed challenges to the therapeutic management of osteosarcoma [142]. Naringin inhibits proliferative and invasive activities and promotes apoptosis in human osteosarcoma cells by suppressing the expression of Zeb1, which is a transcriptional factor involved in tumor metastasis. In an in vivo study, naringin reduced the formation of tumor nodules in a liver injected with MG63 cells [64] (Table 1). Vascular cell adhesion molecule-1 (VCAM-1) is also associated with tumor metastasis. Naringin also suppresses the migration and invasion of malignant cartilaginous neoplasms, as evidenced in the human chondrosarcoma JJ012 and SW1353 cells by increasing the expression of miR-126 and decreasing the expression of VCAM-1. However, naringin does not affect the cell death of human chondrosarcoma, which is the second most malignant bone tumor and accounts for 10–15% of all primary bone tumors [65,143] (Table 1). There is not a great deal of information on the protective activity of naringin against bone and cartilage tumors. More efforts are still needed to elucidate the potential mechanism of naringin in regulating the biological actions of tumor cells.

## 8. The Protective Activities of Naringin against Tibial Dyschondroplasia

Tibial dyschondroplasia (TD) is characterized by ambulation dysfunction, growth retardation, tibial bone deformation, and avascular nonmineralized growth plates. Ihh increases the maturation and proliferation of chondrocytes, while PTHrP suppresses chondrocyte maturation and proliferation in endochondral ossification. The dysregulation of the Ihh/PTHrP axis is closely associated with various bone diseases [144]. In thiram-induced TD broiler chickens, naringin, at a dose of 30 mg/kg, can effectively improve the thiram-induced pathological changes by decreasing Ihh expression and increasing PTHrP expression. Specifically, naringin can increase growth performance, recuperate growth plate width, improve functions, and enhance the levels of antioxidant enzymes [66] (Table 1).

## 9. Conclusions and Future Directions

Naringin exhibits promising therapeutic effects against bone and cartilage disorders, including OP, OA, IDD, RA, and FH diseases, bone and cartilage tumors, and TD. Naringin exhibits osteoprotective effects against OP both in vivo and in vitro by inducing osteogenic differentiation and inhibiting osteoclast formation. The potential molecular mechanisms of naringin in the bone system might be associated with the upregulation of BMP-2 and Wnt/β-catenin as well as the angiogenesis and downregulation of oxidative stress and inflammation. Numerous studies on the potential biomaterial-based applications of naringin have been explored, aiming to decrease its degradation and sustain its release. In addition, naringin also exhibits protective activities against IDD, OA, and RA by ameliorating inflammatory responses and oxidative stress and suppressing cell apoptosis and ECM degradation.

Naringin can be beneficial as a medication for bone and cartilage disorders due to its ability to mediate several signaling pathways. However, naringin has yet to obtain approval as a single or combinational agent for therapeutic use in treating bone and cartilage disorders in the clinic. The potential drug targets of naringin should be demonstrated. Most studies on naringin are investigated in vitro or in animals. Continued efforts to improve bioavailability, pharmacokinetic profiles, and bone/cartilage-target drug delivery systems are still needed. It is essential to elucidate the potential biological functions of naringin in clinical practice.

## Figures and Tables

**Table 1:** The protective activities of naringin in bone and cartilage disorders.

Models/Cells	Doses/Concentrations	Biological Actions	Ref.
ReA-induced rat OP	50 mg/kg	Increases BMD of femur shaft, increases BV/TV and Tb.Sp, increases ALP activity, decreases SOST, TRAP, and RANKL expression, and increases PTH1R expression.	[50]
IDG-SW3 cells	50 μM	Decreases SOST and RANKL expression.	[50]
OVX-induced rat OP	40, 100, 200 mg/kg	Improves BMD, bone indices, and pathological changes. Increases the average maximum fracture load. Increases the serum OC and decreases the serum CTX-1.	[51]
Osteoclasts	20 ng/mL	Reduces the number of TRAP-positive osteoclasts; promotes cell apoptosis.	[51]
OVX-induced rat OP	300 mg/kg + treadmill exercise	Increases bone indices, BMD, and mechanical strength; increases OCN expression and decreases CTX-1 expression.	[52]
GC-treated IBD rats	100 and 200 mg/kg	Decreases the serum TNFα and increases the serum P1NP; improves the bone indices; decreases MDA, CAT, and SOD activity; increases ALP, OC, and RUNX-2 expression.	[53]
NP cells from patients with IDD	20 μg/mL	Increases cell proliferation, increases BMP2, Sox6, and aggrecan expression, and decreases MMP-3 expression.	[54]
IL-1β-treated human NP cells	0.4, 0.8, 1.2, and 1.6 μM	Decreases MMP-3, MMP-13, ADAMTS4, and ADMATS5 expression, increases collagen II and aggrecan production; attenuates p65 and IκBα phosphorylation; decreases p65 and p53 expression.	[55]
TNFα-treated NP cells	10 μg/mL	Decreases COX-2, cleaved caspase-3, Bax, MMP-3, ADAMTS4, and p63 expression; enhances SOD, Bcl-2, collagen II, aggrecan, Sox9, LC3-II/I ratio, AMPK, and Sirt1 expression; improves mitochondrial functions.	[56]
H_2_O_2_-treated NP-derived MSCs	10 μM	Inhibits apoptosis; improves mitochondrial functions; decreases Bax, caspase-3, and p53 expression; increases Bcl-2, PI3K, and AKT expression	[57]
TNFα-treated mouse chondrocytes	5 μM	Decreases IL-1β, iNOS, COX-2, MMP-13, ADAMTS5, p-IκBα, and NF-κB2 expression.	[58]
ACLT-induced mouse OA	100 mg/kg	Decreases IL-1β, iNOS, COX-2, MMP-13, ADAMTS5, p-IκBα, and NF-κB2 expression.	[58]
MIA-induced rat OA	5 and 10 mg/kg	Decreases the serum levels of PGE2, IL-6, IL-1β, and TNFα; improves histopathological changes.	[59]
LPS-treated RAW 264.7 cells	5 and 10 μg/mL	Decreases the production of PGE2, NO, IL-6, and TNFα.	[59]
Cartilage defects in New Zealand rabbits	84 mg/kg + ADM	Improves the repair morphology of defect cartilages and enhances the expression of TGFβ2, TGFβ3, and SOX9.	[60]
TNFα-treated RA FLSs	20, 40, 60, and 80 μg/mL	Decreases cell viability, increases apoptosis, downregulates the expression of IL-1, IL-6, IL-8, MMP-1, MMP-2, and MMP-13, and suppresses the MAPK/ERK and PI3K/AKT signaling pathways.	[61]
MPS-induced rat SANFH	5, 10, and 20 mg/kg	Increases the serum OC levels, decreases the total cholesterol, the LDL/HDL ratio, and caspase-3 expression; promotes osteogenic differentiation by increasing the expression of PPARγ, Notch, β-catenin, and p-AKT.	[62]
Dex-treated MLO-Y4, MC3T3-E1, and RAW 264.7 cells	100 μM	Attenuates cell apoptosis and decreases Bax and cleaved caspase-3 expression; increases Bcl-2 expression; promotes osteogenic differentiation and inhibits osteoclast formation by activating the PI3K/AKT signaling pathway.	[63]
MPS-induced rat GIONFH	300 mg/kg	Improves histopathological changes and enhances the expression of OC and AKT	[63]
MG63 cells	10 and 20 μmol/L	Decreases the expression of Cyclin D1, MMP2, and Bcl-2 and inhibits cell proliferation, migration, and invasion.	[64]
JJ012 and SW1353 cells	3, 10, and 30 μM	Decreases VCAM-1 expression, increases miR-126 expression, and suppresses cell migration and invasion.	[65]
Thiram-induced TD broiler chickens	30 mg/kg	Restores the tibia weight and length, inhibits the reduction in blood vessels, and increases the expression of Ihh and PTHrP.	[66]

## Data Availability

The data used to support the findings of this study are included within the article.

## Abbreviations

OP, osteoporosis; OA, osteoarthritis; IDD, intervertebral disc degeneration; RA, rheumatoid arthritis; FH, femoral head; TD, tibial dyschondroplasia; MSCs; mesenchymal stromal stem cells; BMD, bone mineral density; NF-κB, nuclear factor-κB; RANKL, receptor activator of nuclear factor kappa-B ligand; PTH1R, parathyroid hormone-1 receptor; HMG-CoA, hydroxy-methylglutaryl coenzyme A; BMP-2, bone morphogenetic protein-2; BMSCs, bone mesenchymal stem cells; PPARγ, peroxisome proliferators-activated receptor γ; Sost, sclerosteosis; RUNX2, Runt-related transcription factor-2; TGFβ1, transforming growth factor β1; Smad: suppressor of mothers against decapentaplegic; CXCL-5, C-X-C motif chemokine-5; Osx, osterix; LPS, lipopolysaccharide; BMPRs, BMP receptors; Foxc2, Forkhead box C2; IHH, Indian hedgehog; SERMS, estrogen and selective estrogen receptor modulators; OC/OCN, osteocalcin; CTX-1, C-terminal telopeptides of type I collagen; VEGF, vascular endothelial growth factor; SOD, superoxide dismutase; GSH, glutathione; CAT, catalase; GPX, glutathione peroxidase; JAK2, Janus-activated kinase 2; STAT3, signal transducer and the activator of transcription 3; MMPs, matrix metalloproteinases; ADAMTSs, a disintegrin and metalloproteinase with thrombospondins; COX-2, cyclooxygenase; AMPK, adenosine 5′-monophosphate (AMP)-activated protein kinase; PGE2, Prostaglandin E2; SOX9, SRY-Box transcription factor 9; VCAM-1, vascular cell adhesion molecule 1.
